# Supplementary material for: The Roles of prM-E Proteins in Historical and Epidemic Zika Virus-mediated Infection and Neurocytotoxicity
Source: Viruses. 2019 Feb 14;11(2):157. doi: 10.3390/v11020157 (PMC6409645; doi:10.3390/v11020157)
Supplement: Supplementary file 1 [file viruses-11-00157-s001.zip › supplementary material/LiG22Viruses_020119_supplementary figure legend.docx]

**Supplementary Figure Legend**

**Figure S1.** Representative pictures of the immunostaining assay to measure caspase 3 cleavages (**A**) as shown in **Figure 2C**.

**Figure S2.** Correlation of viral attachment and viral infection between chimeric viruses and their parental viruses in A549 cells. (**A**) Viral binding to SNB-19 cells was measured by presence of cell-associated vRNA one-hour p.i*.*. Results represent average and standard deviation (X + SD) of from three different experiments. (**B**) ZIKV viral replication was measured by plaque formation assay with timeframe as indicated. The input viruses were 5 x 10^7^, 5 x 10^6^, 5 x 10^6^, and 4 x 10^6^ PFU/mL, respectively.

**Figure S3.** Effect of Adv-prM expression on ZIKV-induced cell viability of different cell lines (**A**). Cell viability was measured by the MTT assay day 3 and 5 p.i.. Cells were infected with Adv-prM with different MOI as indicated. SNB-19, human brain glioblastoma cells; HEMEC, human brain microvascular endothelial cell line; and SH-SY5Y, human neuroblastoma cells. The level of cell survival was calculated by subtracting the basal effect of Adv-GFP control. A trending line is added to results of each cell line indicating dose-dependent cell viability.
