# Supplementary material for: The Roles of prM-E Proteins in Historical and Epidemic Zika Virus-mediated Infection and Neurocytotoxicity
Source: Viruses. 2019 Feb 14;11(2):157. doi: 10.3390/v11020157 (PMC6409645; doi:10.3390/v11020157)
Supplement: Supplementary file 1 [file viruses-11-00157-s001.zip › supplementary material/LiG22Viruses_Supplementary Figs_020119.pptx]

## Slide 1
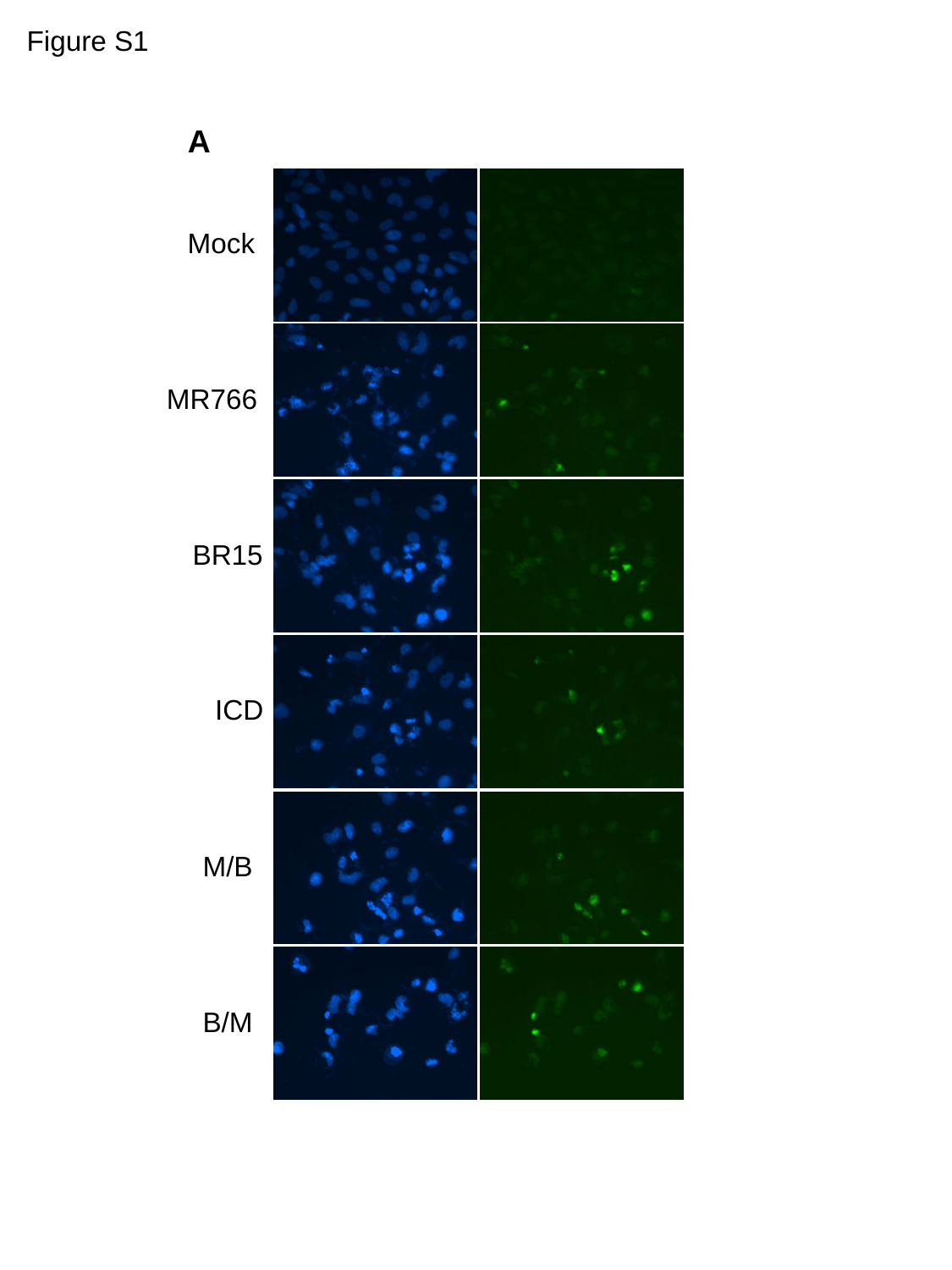

Figure S1
A
Mock
MR766
BR15
ICD
M/B
B/M

## Slide 2
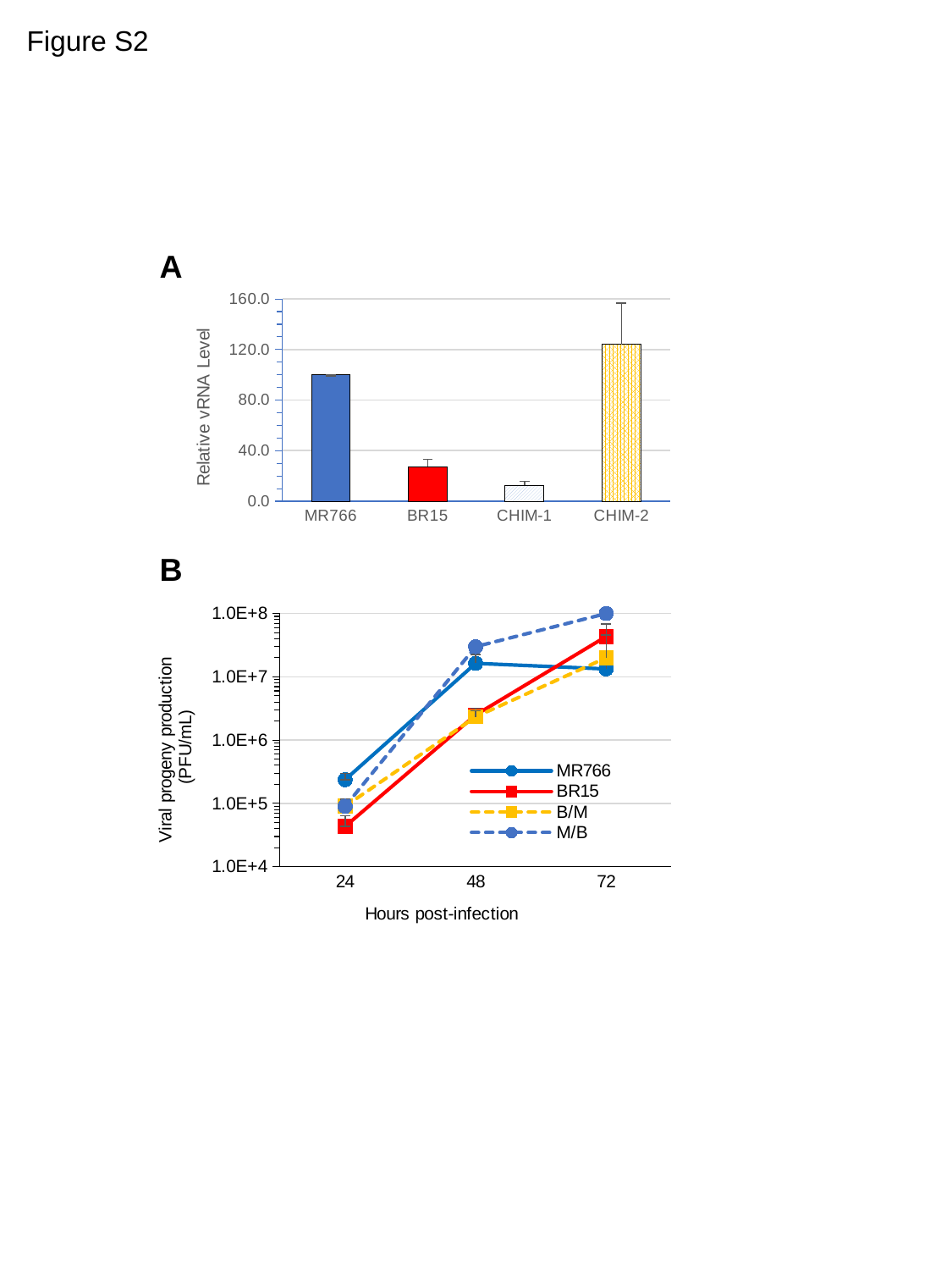

Figure S2
A
### Chart
| Category | |
|---|---|
| MR766 | 100.0 |
| BR15 | 27.3333333333333 |
| CHIM-1 | 12.6666666666667 |
| CHIM-2 | 124.0 |B
### Chart
| Category | MR766 | BR15 | B/M | M/B |
|---|---|---|---|---|
| 24 | 236666.66666666666 | 43333.333333333336 | 90000.0 | 90000.0 |
| 48 | 16333333.333333334 | 2500000.0 | 2333333.3333333335 | 30000000.0 |
| 72 | 13333333.333333334 | 43333333.333333336 | 20000000.0 | 100000000.0 |

## Slide 3
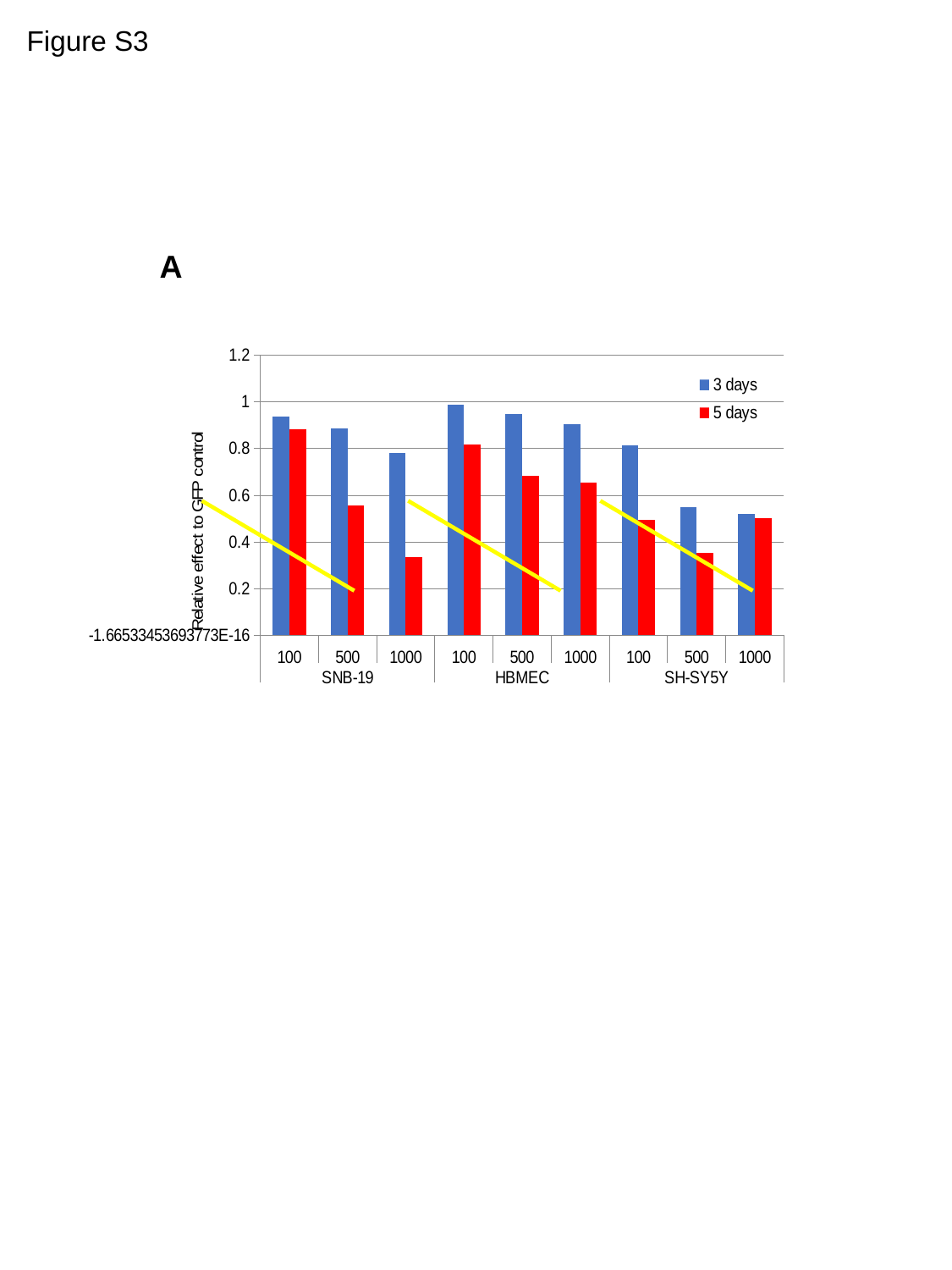

Figure S3
A
### Chart
| Category | 3 days | 5 days |
|---|---|---|
| 100 | 0.9375203384315002 | 0.8830049261083744 |
| 500 | 0.8854844887016469 | 0.5577047066408768 |
| 1000 | 0.7805544709098008 | 0.33357771260997066 |
| 100 | 0.9868114817688131 | 0.8180349932705249 |
| 500 | 0.9494712103407755 | 0.6836105976586567 |
| 1000 | 0.9042904290429045 | 0.6528861154446177 |
| 100 | 0.8146473779385173 | 0.4929494712103408 |
| 500 | 0.549936788874842 | 0.3537604456824513 |
| 1000 | 0.5197628458498024 | 0.5028571428571429 |
